# “Ghost”, a Well-Known but Not Fully Explained Echocardiographic Finding during Transvenous Lead Extraction: Clinical Significance

**DOI:** 10.3390/ijerph191912542

**Published:** 2022-10-01

**Authors:** Dorota Nowosielecka, Wojciech Jacheć, Anna Polewczyk, Łukasz Tułecki, Paweł Stefańczyk, Andrzej Kutarski

**Affiliations:** 1 Department of Cardiology, The Pope John Paul II Province Hospital, 22-400 Zamość, Poland; 2 Department of Cardiac Surgery, The Pope John Paul II Province Hospital, 22-400 Zamość, Poland; 3 2nd Department of Cardiology, Faculty of Medical Sciences, Silesian Medical University, 41-800 Zabrze, Poland; 4 Institute of Medical Sciences, Jan Kochanowski University, 25-369 Kielce, Poland; 5 Department of Cardiac Surgery, Świętokrzyskie Cardiology Center, 25-736 Kielce, Poland; 6 Department of Cardiology, Medical University, 20-059 Lublin, Poland

**Keywords:** transvenous lead extraction, transoesophageal echocardiography, scar tissue, ghost

## Abstract

“Ghosts” are fibrinous remnants that become visible during transvenous lead extraction (TLE). Methods: Data from transoesophageal echocardiography-guided TLE procedures performed in 1103 patients were analysed to identify predisposing risk factors for the development of so-called disappearing ghosts—flying ghosts (FG), or attached to the cardiovascular wall—stable ghosts (SG), and to find out whether the presence of ghosts affected patient prognosis after TLE. Results: Ghosts were detected in 44.67% of patients (FG 15.5%, SG 29.2%). The occurrence of ghosts was associated with patient age at first system implantation [FG (OR = 0.984; *p* = 0.019), SG (OR = 0.989; *p* = 0.030)], scar tissue around the lead (s) [FG (OR = 7.106; *p* < 0.001, OR = 1.372; *p* = 0.011), SG (OR = 1.940; *p* < 0.001)], adherence of the lead to the cardiovascular wall [FG (OR = 0.517; *p* = 0.034)] and the number of leads [SG (OR = 1.450; *p* < 0.002). The presence of ghosts had no impact on long-term survival after TLE in the whole study group [FG HR = 0.927, 95% CI (0.742–1.159); *p* = 0.505; SG HR = 0.845, 95% CI (0.638–1.132); *p* = 0.265]. Conclusions: The degree of growth and maturation of scar tissue surrounding the lead was the strongest factor leading to the development of both types of ghosts. The presence of either form of ghost did not affect long-term survival even after TLE indicated for infection.

## 1. Introduction

The constant interaction between intracardiac leads and the vein and heart structures results in a pathological build-up of the connective tissue around the leads. Lead-related endothelial trauma causes an inflammatory response of the vessel wall with subsequent scarring [1,2,3,4]. Fibrous connective tissue around the lead is well seen with transoesophageal echocardiography (TEE) or intracardiac echocardiography (ICE) [5]. It may have different forms such as floating connective tissue surrounding the lead, mobile thrombi on the lead, vegetation-like masses, lead thickening, adherence of the lead to the vein, cardiac wall, tricuspid apparatus or another lead [6,7,8,9,10,11,12]. Transvenous lead extraction (TLE) is a first-line strategy for the management of lead-related complications associated with cardiac-implantable electronic devices (CIED) [13,14,15]. The reactive fibrous capsule identified by ultrasounds may be a predictor of a more difficult and complicated TLE procedure. Routine post-operative TEE directed our attention to “landscape after battle” not only in search of procedure-related complications but also different forms of residual scar tissue after lead removal [5,8,16,17,18,19]. These tissue remnants have been referred to as “ghosts” [5,16,17,18,19,20,21,22,23,24,25,26,27]. The clinical significance of “ghosts” has been subject to considerable discussion [5,16,17,18,19], but there has been little agreement on their prognostic value, especially in patients with CIED infection.

## 2. Materials and Methods

### 2.1. Study Population

**Figure 1:**
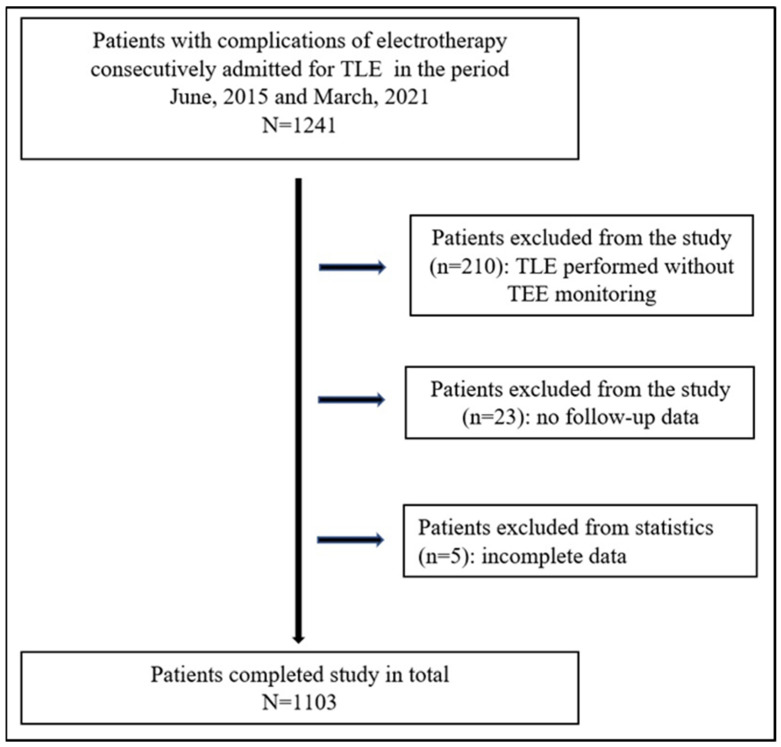
Flow chart of the recruitment process towards the study group.

This post hoc analysis used clinical data of 1103 patients who underwent TLE with complete TEE monitoring between June 2015 and March 2021 at one high-volume centre (Figure 1).

### 2.2. TLE Indications

Indications for TLE were: infectious (pocket infection, bacteremia with or without endocarditis, or any combination of the above) and non-infectious, i.e., mechanical damage of lead (electrical failure), dislodgement, extracardiac pacing, exit/entry block, perforation, upgrading, downgrading, avoidance of lead abandonment, threatening or potentially threatening lead (free ending, left heart, known lead-related TV dysfunction) and other (cancer, painful pocket, MRI and lead extraction to regain venous access in cases of complete venous occlusion including SVC syndrome).

### 2.3. TEE Monitoring of the Lead Extraction Procedure

TEE monitoring was performed using commercially available equipment (GE Vivid S 70 and Philips iE33) connected to a multiplane transducer (probes: 6VT-D, X7-2t Live 3D). All images were stored in a digital memory. In the preprocedural phase we evaluated lead position, extent of fibrous encapsulation, lead-to-lead adhesions, unaccounted-for masses on the leads as well as function of the tricuspid valve (TV) and the pericardium. During the intraprocedural phase TEE allowed monitoring of direct pulling on the cardiac structures, detachment and dislodgement of fibrous tissue or vegetation fragments, and separation of pericardial layers. The postprocedural phase included evaluation of TV function, remnants of masses removed during TLE and abnormal accumulation of fluid in the pericardial sac. Continuous TEE monitoring was described in detail in our earlier reports.

### 2.4. Definitions of Echocardiographic Phenomena

Lead fibrotic attachment to the cardiac wall was defined at TEE either as sleeve-like dense echoes involving the leads and extending to the surrounding structures (TV, atrial and ventricular wall) with loss of independent lead motion. The occurrence of such preoperative phenomena was described in our previous reports [6,7,8,16,28,29] and was not examined in this study. Vegetations identified with TEE before, during and after TLE (vegetation remnants) were not considered as ghosts and were subject to separate classification.

**Figure 2:**
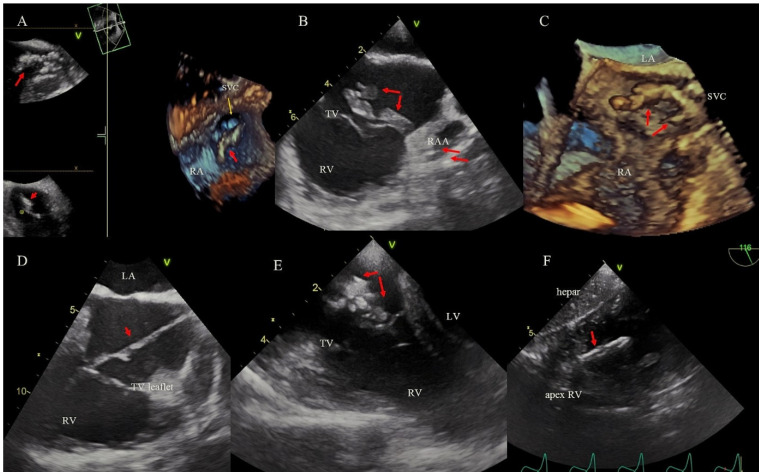
SG (red arrows) in different areas of the cardiovascular system imaged by 2D and 3D TEE. (**A**) SG at the SVC orifice. (**B**) SG leaving the RAA. (**C**) SG in the SVC/RA. (**D**) Long SG stretched between the TV and SVC. (**E**) Large SG in the RA adhering to the septal leaflet. (**F**) SG adhering to the RV endocardium.

Stable ghosts (SG) were defined as new, post-extraction, floating masses visualized during intra- and post-extraction TEE monitoring. They were seen as echogenic, tubular masses with one ending fixed to venous or cardiac walls and structures, and with the second ending floating free (Figure 2), persisting until oesophageal probe removal [16].

**Figure 3:**
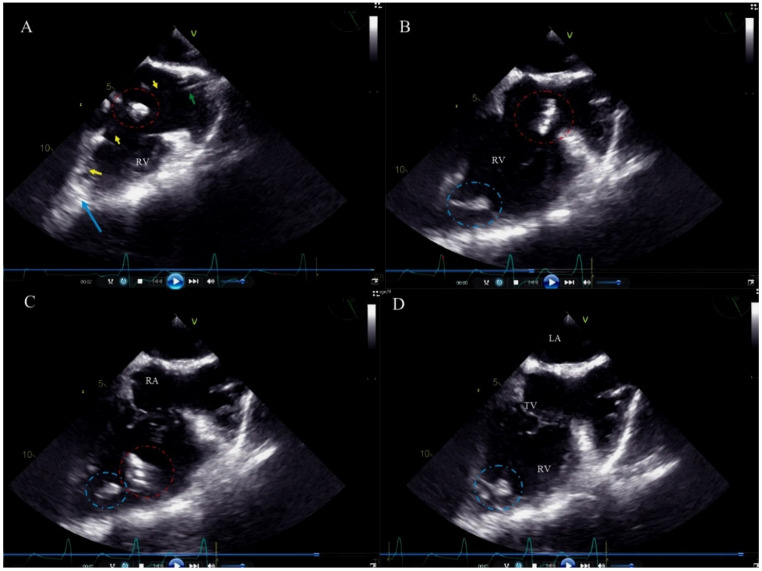
Concomitant occurrence of FG and SG and their fate imaged by 2D TEE (the sequences of the video and the behavior of the ghosts are represented “frame by frame”). (**A**) Ventricular lead (yellow arrows) dissected using the Byrd dilator sheath (green arrow). Scar tissue pushed in front of the catheter (red circle). The end piece of the lead adhered to the RA endocardium (blue arrow). (**B**) After lead extraction FG (red circle) in the RA and SG (blue circle) in the RV. (**C**) FG travelled in the bloodstream to the RV in the next heartbeat, whereas SG remained in the RA. (**D**) In the subsequent cardiac cycles FG „disappeared” in the pulmonary circulation, only SG remained in place.

Flying ghosts (FG) represent a dynamic phenomenon which may be observed while freeing the lead and cutting off the encapsulating fibrous tissue. When the dilatating sheath is moved down, the masses remain attached to the cardiovascular structures or are freed and after making several circular motions, flow into the TV, right ventricle (RV), thus disappearing from the TEE field of view. The phenomenon of FG was considered occurring when the described sequence of events was documented (Figure 3).

**Figure 4:**
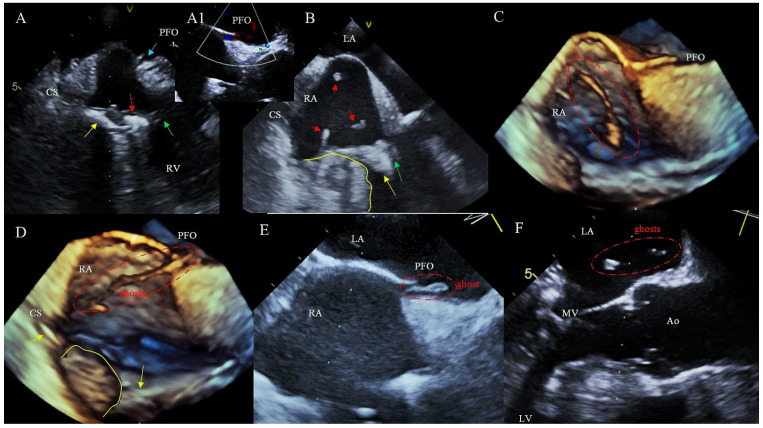
Flying ghosts that travelled to the systemic circulation through the PFO imaged by 2D, 3D TEE (**A**) Coronary sinus lead extraction (yellow arrow) (CS). Encapsulating scar tissue (red arrow) dissected away from the pacing lead using the Byrd dilator sheath (green arrow). (**B**) Pieces of scar tissue (FG) (red arrow) freed during lead dissection. Pulling on the cardiac wall (yellow line) during extraction of the adherent lead. (**C**) FG, freed from the lead, floating in the RA. (**D**,**E**) The same FG passing through the PFO. (**F**) FG in the LA.

There was also a rare case of an FG that travelled to the systemic circulation through a patent foramen ovale (PFO) (Figure 4).

**Figure 5:**
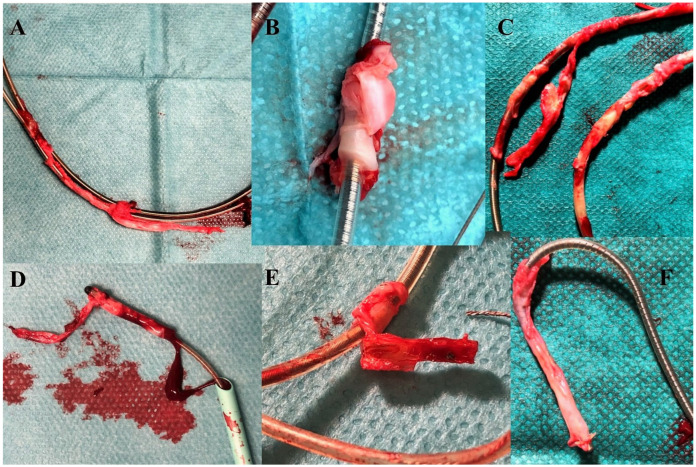
Examples of scar tissue surrounding the leads as seen after extraction (**A**,**C**,**D**,**F**) Examples of scar tissue surrounding the leads as seen after extraction (**B**) Calcification of the scar tissue. (**E**) Ossification of the scar tissue.

It should be underlined that scar tissue surrounding the lead is not visualized if located in the anonymous and subclavian veins. During mechanical lead dissection the encapsulation may be covered with a larger dilatating sheath and removed together with the lead (Figure 5).

Unlike mechanical systems, excimer laser sheaths utilise laser energy to vaporize fibrous tissue, thus decreasing ghost detection.

### 2.5. Lead Extraction Procedure

Lead extraction procedures were performed in a hybrid operating room or in an operating room, using mechanical systems such as polypropylene Byrd dilator sheaths (Cook^®^ Medical, Leechburg, PA, USA), making use of the oblique cutting edge of the tip to dissect leads from fibrous sheaths that immobilized the intravascular and/or intracardiac segment of the lead [13,14,15]. Complete procedural, clinical success and complications of TLE were defined according to the HRS 2009 and 2017 guidelines and the 2018 EHRA expert consensus statement [13,14,15]. Major and minor complications were defined according to the 2018 EHRA Expert Consensus Statement on Lead Extraction [15].

### 2.6. Approval of the Bioethics Committee

The study was conducted according to the ethical guidelines of the Declaration of Helsinki and approved by the Bioethics Committee at the Regional Chamber of Physicians in Lublin no. 288/2018/KB/VII.

### 2.7. Statistical Analysis

The Shapiro–Wilk test was used to check whether continuous variables followed a normal distribution. Despite nonparametric distribution of some data (most continuous variables were normally distributed), for uniformity, all continuous variables are presented as the mean ± standard deviation. The categorical variables are presented as number and percentage. The significance of differences between groups (1,2,3) was determined using the nonparametric Chi^2^ test with Yates correction or the unpaired Mann–Whitney U test, as appropriate. Univariate and multivariable logistic regression was used to determine which parameters affected the occurrence of ghosts. The variables achieving *p* < 0.1 were included in the multivariable linear regression analysis. To determine the impact of FG and SG on long-term survival after TLE uni- and multivariable Cox regression analysis was used. The variables achieving *p* < 0.1 under univariable regression analysis were included in the multivariable regression model. To illustrate the impact of FG and SG on survival, the Kaplan–Meier survival curves were constructed and evaluated with log rank test. A *p* value of 0.05 or less was considered statistically significant. Statistical analyses were performed using Statistica version 13.3 (TIBCO Software Inc., Palo Alto, CA, USA).

## 3. Results

Baseline demographic and clinical characteristics of the study groups are summarized in Table 1.

TLE was indicated for systemic infection in 174 pts (15.78%), local (pocket) infection in 66 pts (5.98%) and non-infectious reasons in 863 cases (78.24%). Most patients (69.54%) had a pacemaker (any), whereas only 8.79% had a CRT-D device. Implant duration expressed as dwell time of the oldest lead in the patient before TLE was 120.5 months (Table S1). The rate of major complications was 2.45%, clinical success was obtained in 98.91% and procedural success in 95.74% of patients (Table S2).

Analysis of the clinical data (Table 1) indicated that only younger age at TLE and age at first system implantation increased the chances for the development of both types of ghosts. FG was more likely to develop in patients with non-infectious indications for extraction of a dual lead pacemaker system. Ghosts after TLE were more frequent in patients with multiple lead systems and numerous leads in the heart, similarly to patients with more CIED-related procedures before lead extraction. The most important factor explaining the presence of FG seem to be implant duration before TLE. In summary, the main factors for developing ghosts were the number of leads in the case of SG, and implant duration but not systemic infection as regards FG (Table S1).

Comparative analysis of TLE-related potential risk factors for major complications, procedure complexity and TLE efficacy (Table S2) showed that extraction of abandoned lead(s) predisposed to ghosts after TLE. The occurrence of any technical problem/difficulty, hemopericardium and TV damage during TLE were much more frequent in patients with FG. However, it seems to be a result of implant duration as well as the extent and maturation of scar tissue surrounding the leads (Tables S2 and S3).

Table S3 summarizes TEE findings before and after TLE with special focus on the presence of all forms of scar tissue. The last rows of the table compare mortality after TLE during short- and long-term follow-up depending on the type of ghost. Generally, patients with scar tissue surrounding the lead, blood clots on the lead, vegetation-like masses, lead thickening, true vegetations, lead adherence to any heart structure and lead-to-lead binding were much more prone to having both forms of ghosts, particularly FG. The more common presence of TLE-related severe TV damage (increased TR by 3 degrees and damage to tendinous chords) in patients with ghosts seemed to be secondary to implant duration or connective tissue growth and maturation.

### 3.1. Uni- and Multivariable Linear Regression Analysis

Univariable analysis showed that the factors predisposing to FG formation were older leads, the number of previous CIED-related procedures, lead-related scar tissue, the number of scars and lead-to-lead binding or adhesion to anatomical structures relating to the cardiovascular system (CVS). FG were less likely to develop in patients with older age at first CIED implantation and with lead-related infective endocarditis as indications for TLE. Multivariable regression analysis showed that any form of scar tissue around the lead and the number of scars were the main factors responsible for FG. Older age at first CIED implantation and the presence of strong lead-to-lead binding or adhesion to CVS structures decreased the probability of FG (Table 2).

Univariate analysis identified the following factors predicting the occurrence of SG after TLE: abandoned lead(s), the number of leads in the heart, scar tissue around the leads and the number of scars as well as lead-to-lead binding and adhesions to CVS structures. Older age at the time of implantation of the first CIED was associated with a lower probability of SG. Multivariable regression analysis showed that the factors increasing the likelihood of SG were the number of the leads in the heart and any form of lead-related scar tissue. Older age at first CIED implantation decreased the probability of SG (Table 2).

### 3.2. Survival Analysis

A total of 281 (25.48%) patients died during 1264 ± 644.7 (2–2466) days of follow-up.

**Figure 6:**
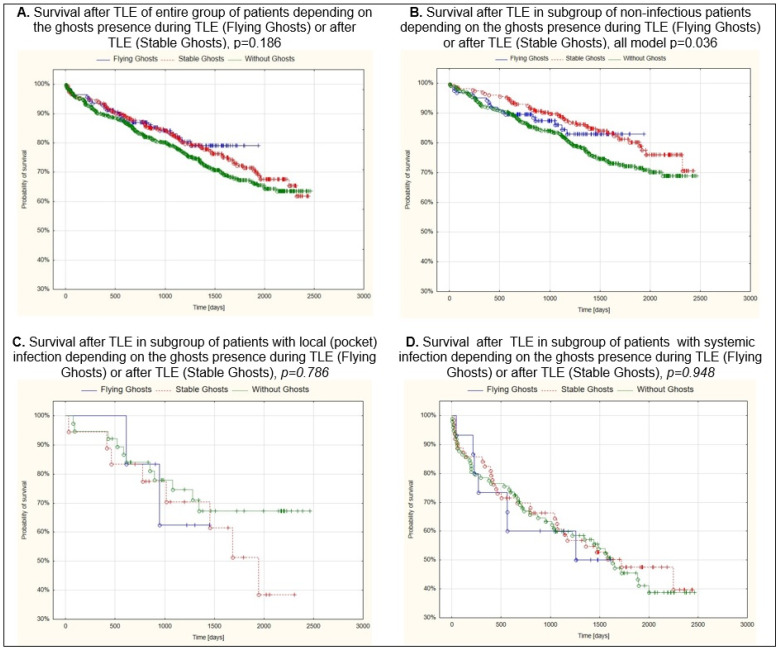
Kaplan–Meier probability of survival after TLE based on the presence of ghosts during or after TLE.

As expected, survival in the group of patients who underwent TLE for infectious reasons was significantly worse compared to the group of patients who underwent TLE for non-infectious reasons. The 30-day mortality was 1.55% and was not related to the presence of both floating and stable ghosts. The log rank analysis of the survival curves of Kaplan–Meier did not show any influence of the presence of both forms of ghosts on long-term survival in the entire group of patients. Only the subgroup of patients with non-infectious indications for TLE showed statistically significantly better survival in whom the presence of stable ghosts after TLE was demonstrated (log rank *p* = 0.034) Table 3, Figure 6.

Multivariable Cox regression analysis confirmed the importance of the common factors that influence patient prognosis after TLE. In the entire group of patients mortality risk increased with older age (HR = 1.049; *p* < 0.001), higher NYHA functional class (HR = 1.462; *p* = 0.002), presence of any renal failure (HR = 1.550; *p* < 0.001), permanent atrial fibrillation (HR = 1.444; *p* = 0.004), higher Charlson comorbidity index (HR = 1.044; *p* = 0.012), resynchronisation therapy before TLE (HR = 1.072; *p* < 0.001), and systemic infection (HR = 1.894; *p* < 0.001). Women (HR = 0.621; *p* < 0.001) and patients with higher LVEF (HR = 0.987; *p* = 0.009) were less likely to die during follow-up.

In the infectious subgroup worse survival was associated with older age, higher NYHA class, presence of any renal failure and resynchronisation therapy before TLE. Prognosis appeared to be better in women and patients with higher LVEF.

In the non-infectious subgroup the prognostic factors were the same as in the entire group of patients (Table 4). Our analysis showed no impact of ghosts on long-term mortality after TLE both in the entire group of patients and in the two subgroups (infectious and non-infectious) (Table 4).

This result was confirmed mostly by the Kaplan-Meier survival curves and log rank analysis (*p* = 0.186 for the entire group) (Figure 6A). However, in the non-infectious subgroup, survival after TLE was significantly better in those with stable ghosts (*p* = 0.014) (Figure 6B), (Table S3).

## 4. Discussion

The most important finding of this study is that the presence of either form of ghost did not affect long-term survival after TLE indicated for device infection (Figure 6C,D).

Remnants of scar tissue anteriorly surrounding the lead before extraction were described in several case reports and named as “ghosts” [21,22,23,24,25,26,27]. Thereafter, a series of six reports were published [5,16,17,18,19], five of them addressed the importance of “stable ghosts” in the aspect of long-term mortality. The phenomenon was observed in 8% [19], 14% [18], 19% [17] and 60% [5] of post-operative TEE images. The reports on TEE monitoring during TLE procedures did not consider analysis of scar tissue remnants or the dynamic phenomenon of scar tissue cut free (mobilization) [28,29,30,31,32]. Out of the five large studies on “ghosts” Poterała et al. did not examine the influence of ghosts on long-term mortality [17], Narducci underlined that the presence of ghosts could be an independent predictor of mortality after TLE in patients with infections [18], Le Dolley indicated that ghosts were suggestive of device infection and seemed to be associated with the diagnosis of lead-related endocarditis [19]. Diemberger concluded that the presence of ghosts at post-TLE transoesophageal echocardiography and a closed CIED pocket were associated with a worse prognosis [20] but Caiati suggested that ghosts were mostly benign remnants of fibrotic lead capsule cut off during extraction [5]. A large sample of patients undergoing TEE during TLE combined with detailed description of various forms of scar tissue prompted us to perform this analysis. The present results show that the number of leads and implant duration, but not infectious indications, were the main predictors of ghost formation after TLE. Such TEE findings as scar tissue surrounding the lead, mobile thrombi, vegetation-like masses, lead thickening, lead adherence to heart structures and lead-to-lead binding were much more frequent in patients with both forms of ghosts, particularly with FG. The occurrence of ghosts had no negative prognostic significance. We described an ephemeral but not rare type of ghost—“flying ghost”—that can be observed during scrupulous TEE examinations in patients undergoing TLE.

Narducci showed that infective endocarditis and higher Charlson comorbidity score [18] were significant predictors of “ghosts”. However, we could not confirm this finding in our study. These differences can be explained in part by the sample size and the rate of systemic infection (64.1% versus 15.8%). In our study, the Charlson comorbidity score had no influence on the incidence of ghosts. TLE in patients with systemic infection carries a risk of septic pulmonary embolism which is related to the size of vegetations [19]. However, assessment of the extent of pulmonary vascular obstruction after TLE was not the goal of our study. According to the results in the present study it can be concluded that the amount of thrombotic material seen as travelling “ghosts” is irrelevant from a pulmonary haemodynamic point of view. This explains no impact on long-term survival.

We are aware that the long-term mortality of patients after TLE may have been influenced by other health-related factors such as the presence and extent of infection, comorbidities, the presence of chronic atrial fibrillation as well as the systemic immune-inflammation reaction [5,8,16,29,33,34]. However, we focused on the relevance of the connective tissue remnants released from the leads during the procedure. A phenomenon that raises disputes as to its prognostic significance.

TEE seems to be an appropriate tool for visualisation of scar tissue around the leads, since various forms of fibrosis were detected in 44% of the study patients. Furthermore, continuous monitoring of the extraction procedure permits visualisation of the fleeting nature of FG. Caiati suggests an even better sensitivity of ICE which can show the presence of SG in 60% of patients during TLE [5]. However, ICE is an invasive procedure involving insertion of an ICE tube. This remains in anatomical conflict with dilating sheaths and requires temporary withdrawal of the tube up to the inferior vena cava, thus limiting observation of mobile masses.

An additional aspect of scar tissue mobilisation during TLE, which is worth mentioning, is the presence of PFO and associated risk of crossed embolism. In our study, PFO was detected in 15 patients, however, only one of them had asymptomatic crossed embolism. The presence of CIED alone does not increase the risk of ischemic stroke in patients with PFO [35]. Paradoxical septic embolism can occur in the concomitant presence of CIED and PFO due to migration of thrombotic material during lead removal. A significant risk of brain emboli is associated with coexistence of vegetations and R-L leakage at any level of the right heart [36]. On the other hand, FG may be related to stroke during and after TLE [18,35]. It can be concluded that screening for PFO before TLE is justifiable. Studies on strategies of lead extraction in patients with a PFO, such as closure of the foramen ovale before TLE or temporary placement of filter devices for cerebral protection are warranted. Particular attention should be paid to patients with CIED infections and extensive build-up of scar tissue around the leads combined with a PFO meeting criteria for paradoxical embolism [7].

## 5. Conclusions

1. In approximately 30% of TLE procedures ghosts remain attached to the CVS wall (SG) but in around 15% of the extraction procedures freed ghosts (FG) travel spontaneously to the pulmonary vascular bed and disappear.
2. Younger patient age and the number of leads but not infectious indications are the factors predisposing to ghost formation during and after TLE.
3. The degree of growth and maturation of the connective tissue surrounding the lead before TLE is the strongest predictor of both types of ghosts.
4. The occurrence of ghosts is associated with complicated procedures, but it seems to be related to implant duration and scar growth.
5. The presence of both types of ghosts does not reduce survival after TLE.

### Study Limitations

This is a single-center, observational, prospective study. TEE monitoring was mandatory. ICE was not the aim of the study. TLE was performed using mechanical systems without laser energy. We cannot interpolate our results on patients in whom laser energy is utilized. Histopathological analysis of “ghost” was not performed. Ghost formation in the anonymous veins was not explored by ultrasounds. The study did not diagnose asymptomatic pulmonary embolism using CT angiography.

## Figures and Tables

**Table 1:** Patient characteristics and comparison of potential patient-related risk factors for the occurrence of both forms of ghost.

Patient Characteristics	Flying Ghosts (Ghosts Shifting Spontaneously to Pulmonary Vascular Bed)	Stable Ghosts (Ghosts Remaining Attached to Cardiovascular Wall)	Ghosts Absent during and after TLE
Group/number of patients	1: N = 171 (15.50%)	2: N = 322 (29.19%)	3: N = 610 (55.31%)
Form of result presentation	mean ± SD/n (%) *p* (1 vs. 2)	mean ± SD/n (%) *p* (2 vs. 3)	mean ± SD/n (%) *p* (1 vs. 3)
Patient age during TLE [years]	66.06 ± 16.20 *p* = 0.912	65.73 ± 15.29 *p* < 0.006	68.87 ± 13.38 *p* < 0.047
Patient age at first system implantation [years]	53.53 ± 18.63 *p* = 0.044	56.21 ± 17.39 *p* = 0.028	59.37 ± 14.81 *p* < 0.001
Female	70 (40.94) *p* = 0.654	140 (43.48) *p* = 0.260	232 (38.03) *p* = 0.267
Etiology: ischemic heart disease	109 (63.72) *p* = 0.990	204 (63.35) *p* = 0.421	404 (66.23) *p* = 0.545
NYHA III & IV	31 (18.13) *p* = 0.799	54 (16.77) *p* = 0.854	98 (16.07) *p* = 0.592
LVEF average [%]	48.43 ± 15.07 *p* = 0.610	49.04 ± 14.86 *p* = 0.335	47.93 ± 15.82 *p* = 0.819
LVEF category: significantly decreased (<30%)	24 (14.04) *p* = 0.714	40 (12.42) *p* = 0.101	102 (16.72) *p* = 0.468
Permanent atrial fibrillation	39 (22.81) *p* = 0.937	71 (22.05) *p* = 0.499	148 (24.26) *p* = 0.770
Congestive heart failure	58 (33.92) *p* = 0.085	84 (26.09) *p* = 0.652	169 (27.71) *p* = 0.137
Renal failure (any)	34 (19.88) *p* = 0.451	75 (23.19) *p* = 0.313	162 (26.56) *p* = 0.377
Charlson comorbidity index	5.175 ±4.185 *p* = 0.710	4.708 ± 3.746 *p* = 0.099	5.090 ± 3.671 *p* = 0.434

TLE—transvenous lead extraction, SD—standard deviation, N number, NYHA—New York Heart Association functional class, LVEF—left ventricular ejection fraction.

**Table 2:** Factors that influenced ghost (flying or stable) occurrence during and after TLE, results of uni- and multivariable regression analysis.

	Univariable Regression			Multivariable Regression		
	OR	95% CI	*p*	OR	95% CI	*p*
Flying ghosts						
Patient age at first system implantation [by year]	0.977	0.967–0.987	<0.001	0.984	0.970–0.997	0.019
Systemic infection	0.526	0.301–0.920	0.024	0.539	0.289–1.006	0.052
Number of CIED-related procedures before TLE	1.227	1.046–1.439	0.012	1.081	0.832–1.403	0.560
Dwell time of the oldest target lead in the patient before TLE [by year]	1.060	1.035–1.085	<0.001	0.988	0.946–1.033	0.607
Any form of scar tissue on the lead(s)	10.34	5.625–19.02	<0.001	7.106	3.424–14.75	<0.001
Number of separate scars	1.685	1.457–1.949	<0.001	1.372	1.076–1.749	0.011
Lead adhesion to heart structures (any)	2.811	1.961–4.028	<0.001	0.517	0.281–0.953	0.034
Stable ghosts						
Patient age at first system implantation [by year]	0.989	0.985–0.993	<0.001	0.989	0.980–0.999	0.030
Charlson comorbidity index	0.976	0.940–1.013	0.197			
Abandoned lead before TLE	1.883	1.207–2.938	0.005	1.152	0.653–2.032	0.623
Number of leads in the heart before TLE	1.554	1.280–1.887	<0.001	1.450	1.141–1.842	0.002
Number of CIED-related procedures before lead extraction	1.127	0.981–1.296	0.092	0.940	0.786–1.124	0.499
All forms of scar tissue on the lead(s)	2.304	1.708–3.106	<0.001	1.940	1.293–2.909	<0.001
Number of separate scars	1.424	1.260–1.609	<0.001	1.147	0.939–1.403	0.179
Lead adhesion to heart structures (any)	1.699	1.252–2.307	<0.001	1.045	0.658–1.660	0.851

TLE—transvenous lead extraction, CIED—cardiac implantable electronic device.

**Table 3:** Long term and 30-d survival after TLE.

Long Term and 30-Days Survival after TLE	Flying Ghosts (Ghosts Shifting Spontaneously to Pulmonary Vascular Bed)	Stable Ghosts (Ghosts Remaining Attached to Cardiovascular Wall)	Ghosts Absent during and after TLE
Group/number of patients	1: N = 171	2: N = 322	3: N = 610
Long-term survival of entire group of patients after TLE during mean 1264 ± 644.7 (2–2466) days follow-up. Log rank *p* for all model = 0.186			
Follow-up (mean ± SD) [days]	1048 ± 417.5 *p* < 0.001	1511 ± 588.0 *p* = 0.567	1544 ± 555.0 *p* < 0.001
Alive during follow-up (entire group) (n, %)	143 (83.63) Log rank test *p* = 0.914	242 (75.16) Log rank test *p* = 0.121	437 (71.64) Log rank test *p* = 0.202
Died during follow-up (entire group) (n, %)	28 (16.37) Log rank test *p* = 0.914	80 (24.84) Log rank test *p* = 0.121	173 (28.36) Log rank test *p* = 0.202
Long-term survival after TLE in the subgroup of non-infectious and infectious patients. Log rank *p* for all model = 0.036			
Non-infectious; died/alive (n, %)	19/131 (12.67) Log rank test *p* = 0.430	41/200 (17.01) Log rank test *p* = 0.014	114/360 (24.05) Log rank test *p* = 0.259
Infectious (all); died/alive (n, %)	9/12 (42,86) Chi2 (vs. non-infectious) *p* = 0.001	39/42 (48.15) Chi2 (vs. non-infectious) *p* < 0.001	59/77 (43.38) Chi2 (vs. non-infectious) *p* < 0.001
Long-term survival after TLE in subgroup of patients with pocket infection. Log rank *p* for all model = 0.786			
Local (pocket) infection; died/alive (n, %)	2/4 (33.33) Log rank test *p* = 1.000	8/10 (44.44) Log rank test *p* = 0.463	11/27 (28.95) Log rank test *p* = 0.917
Long term survival after TLE in subgroup of patients with systemic. Log rank *p* for all model = 0.948			
Systemic infection; died/alive (n, %)	7/8 (46.67) Log rank test *p* = 0.789	31/32 (49.21) Log rank test *p* = 0.839	48/50 (48.98) Log rank test *p* = 0.812
30-days survival after TLE in entire group of patients			
Entire group; died/alive (n, %)	2/169 (1.17) Chi2 (1 vs. 2) *p* = 0.660	7/315 (2.17) Chi2 (2 vs. 32) *p* = 0.747	10/600 (1.64) Chi2 (1 vs. 3) *p* = 0.929
30-days survival after TLE in subgroup of non-infectious patients			
Non-infectious; died/alive (n, %)	2/148 (1.33) Chi2 (1 vs. 2) *p* = 0.972	2/239 (0.83) Chi2 (2 vs. 3) *p* = 0.679	4/470 (0.84) Chi2 (1 vs. 3) *p* = 0.956
Infectious (all); died/alive (n, %)	0/21 (0.00) Chi2 (vs. non-infectious) *p* = 0.581	5/76 (6.17) Chi2 (vs. non-infectious) *p* = 0.016	6/130 (4.41) Chi2 (vs. non-infectious) *p* = 0.012
30-days survival after TLE in subgroup of patients with pocket infection			
Local (pocket) infection; died/alive (n, %)	0/6 (0.00)	0/18 (0.00)	0/38 (0.00)
Long term survival after TLE in subgroup of patients with systemic infection			
Systemic infection; died/alive (n, %)	0/15 (0.00) Chi2 (1 vs. 2) *p* = 0.588	5/58 (7.94) Chi2 (2 vs. 3) *p* = 0.900	6/92 (6.12) Chi2 (1 vs. 3) *p* = 0.714

TLE—transvenous lead extraction, SD—standard deviation.

**Table 4:** Factors influencing long-term prognosis after TLE, results of uni- and multivariable Cox regression analysis.

	Univariable Cox Regression			Multivariable Cox Regression		
	HR	95%	*p*	HR	95%	*p*
All patients						
Female gender	0.441	0.336–0.579	<0.001	0.621	0.463–0.833	<0.001
Patient age during TLE [by year]	1.049	1.038–1.061	<0.001	1.040	1.025–1.054	<0.001
NYHA FC class [by one]	2.914	2.410–3.523	<0.001	1.526	1.197–1.946	<0.001
LVEF [by 1p%]	0.966	0.958–0.973	<0.001	0.987	0.981–0.978	0.009
Renal failure (any)	3.269	2.587–4.131	<0.001	1.599	1.243–2.058	0.001
Ischemic heart disease	1.457	1.125–1.887	0.004	1.014	0.758–1.355	0.927
Permanent atrial fibrillation	2.372	1.866–3.015	<0.001	1.443	1.122–1.856	0.004
Charlson comorbidity index	1.142	1.111–1.174	<0.001	1.044	1.010–1.080	0.012
Flying ghosts (1st group)	0.795	0.537–1.178	0.252			
Stable ghosts (2nd group)	0.881	0.680–1.141	0.336			
Ghosts absent (3rd group)	1.223	0.962–1.555	0.100			
ICD presence before TLE	1.156	0.884–1.512	0.292			
Device type: CRTP/CRTD	3.125	2.382–4.100	<0.001	1.702	1.248–2.322	<0.001
Systemic infection	2.854	2.220–3.667	<0.001	1.894	1.460–2.457	<0.001
Isolated pocket infection	1.281	0.829–1.979	0.265			
Infectious patients						
Female gender	0.485	0.301–0.780	0.003	0.578	0.347–0.964	0.036
Patient age during TLE [by year]	1.027	1.009–1.045	0.003	1.037	1.014–1.061	0.002
NYHA FC class [by one]	2.278	1.687–3.075	<0.001	1.659	1.118–2.462	0.012
LVEF [by 1p%]	0.969	0.957–0.981	<0.001	0.988	0.972–1.004	0.133
Renal failure (any)	2.512	1.729–3.649	<0.001	1.686	1.136–2.503	0.009
Ischemic heart disease	0.938	0.620–1.421	0.764			
Permanent atrial fibrillation	1.642	1.106–2.438	0.014	1.010	0.657–1.552	0.965
Charlson comorbidity index	1.096	1.047–1.147	<0.001	1.025	0.972–1.082	0.363
Flying ghosts (1st group)	1.089	0.548–2.163	0.807			
Stable ghosts (2nd group)	1.041	0.705–1.536	0.841			
Ghosts absent (3rd group)	0.940	0.645–1.369	0.747			
Device type: ICD	1.018	0.666–1.556	0.935			
Device type: CRTP/CRTD	2.783	1.861–4.161	<0.001	1.766	1.087–2.868	0.022
Isolated pocket infection	0.560	0.351–0.894	0.015	1.083	0.581–2.018	0.801
Vegetations presence	1.620	1.094–2.399	0.016	1.405	0.835–2.365	0.201
Non-infectious patients						
Female gender	0.478	0.342–0.669	<0.001	0.638	0.443–0.919	0.016
Patient age during TLE [by year]	1.056	1.040–1.071	<0.001	1.043	1.024–1.062	0.000
NYHA FC class [by one]	3.140	2.439–4.043	<0.001	1.619	1.165–2.249	0.004
LVEF [by 1p%]	0.965	0.956–0.975	<0.001	0.988	0.975–1.000	0.048
Renal failure (any)	3.356	2.483–4.535	<0.001	1.489	1.072–2.068	0.017
Ischemic heart disease	1.602	1.151–2.230	0.005	0.882	0.618–1.258	0.488
Permanent atrial fibrillation	2.815	2.078–3.814	<0.001	1.693	1.236–2.320	0.001
Charlson comorbidity index	1.148	1.108–1.190	<0.001	1.058	1.013–1.105	0.011
Flying ghosts (1st group)	0.822	0.509–1.330	0.425			
Stable ghosts (2nd group)	0.721	0.508–1.024	0.068	1.103	0.633–1.923	0.728
Ghosts absent (3rd group)	1.428	1.043–1.954	0.026	1.352	0.821–2.226	0.236
Device type: ICD	1.162	0.820–1.645	0.398			
Device type: CRTP/CRTD	2.758	1.895–4.015	<0.001	1.684	1.126–2.520	0.011

TLE—transvenous lead extraction, NYHA FC—New York Heart Association functional class, LVEF—left ventricular ejection fraction, ICD—implantable cardioverter defibrillator, CRTP—cardiac resynchronisation therapy pacemaker, CRTD—cardiac resynchronisation therapy defibrillator.

## Data Availability

Readers can access data supporting the conclusions of the study upon reasoned request to the authors.

## Supplementary Materials

The following supporting information can be downloaded at: https://www.mdpi.com/article/10.3390/ijerph191912542/s1, Table S1. Indications for TLE, existing CIED and history of pacing. Table S2. TLE-related potential risk factors for major complications, procedure complexity and TLE efficacy and complications. Table S3. TEE findings during TLE with special focus on the presence all forms of scar tissue and mortality after TLE.
