# Supplementary material for: “Ghost”, a Well-Known but Not Fully Explained Echocardiographic Finding during Transvenous Lead Extraction: Clinical Significance
Source: Int J Environ Res Public Health. 2022 Oct 1;19(19):12542. doi: 10.3390/ijerph191912542 (PMC9565986; doi:10.3390/ijerph191912542)
Supplement: Supplementary file 1 [file ijerph-19-12542-s001.zip › ijerph-1937770-supplementary.pdf]

Table S1: Indications for TLE, existing CIED and history of pacing.

Indications for TLE, existing CIED and history of pacing	Flying ghosts (Ghosts shifting spontaneously to pulmonary vascular bed)	Stable ghosts (Ghosts remaining attached to cardiovascular wall)	Ghosts absent during and after TLE
Group / number of patients	1: N=171	2: N=322	3: N=610
Forms of results presentation	mean ± SD / n (%)<br>P (1 vs. 2)	mean ± SD / n (%)<br>P (2 vs. 3)	mean ± SD / n (%)<br>P (1 vs. 3)
TLE indications			
Systemic infection	15 (8.77)<br>P=0.004	62 (19.26)<br>P=0.229	97 (15.90)<br>P=0.026
Local (pocket) infection	6 (3.51)<br>P=0.349	19 (5.90)<br>P=0.730	41 (6.72)<br>P=0.159
Noninfectious indications (lead dysfunction, change of mode pacing, abandoned lead, threatening / potentially threatening lead, MRI indication, cancer, painful pocket, loss of indications for pacing / ICD), regaining venous access)	150 (87.72)<br>P<0.001	241 (74.84)<br>P=0.432	472 (74.84)<br>P=0.004
Lead dysfunction (any reason)	98 (57.31)<br>0.890	181 (56.22)<br>0.987	344 (56.39)<br>0.899
Other indications (change of mode pacing, abandoned lead, threatening / potentially threatening lead, MRI indication, cancer, painful pocket, loss of indications for pacing / ICD), regaining venous access)	52 (30.41)<br>P=0.004	60 (18.63)<br>P=0.0.595	124 (20.65)<br>P=0.007
Change of pacing mode/ upgrading, downgrading	24 (14.04)<br>P=0.004	19 (5.90)<br>P=0.730	41 (6.72)<br>P=0.004
Other indications (abandoned lead, threatening / potentially threatening lead, MRI indication, cancer, painful pocket, loss of indications for pacing / ICD), regaining venous access)	28 (16.37)<br>P=0.331	41 (12.73)<br>P=0.786	83 (13.93)<br>P=0.428
Existing CIED and history of pacing			
Device type – PM: AAI,VVI,VDD (single lead)	34 (19.88)<br>P=0.017	37 (11.49)<br>P<0.001	143 (23.44)<br>P=0.379
Device type – PM: DDD (dual lead)	82 (47.95)<br>P<0.001	181 (56.21)<br>P<0.001	259 (42.46)<br>P=0.233
Device type - CRT-P (three leads)	9 (5.26)<br>P=0.115	7 (2.17)<br>P=0.846	13 (2.13)<br>P=0.054
Device type - ICD (VVI, DDD)	32 (18.71)<br>P=0.849	64 (19.88)<br>P=0.245	143 (23.44)<br>P=0.227

Device type - CRT-D	13 (7.60)<br>P=0.474	33 (10.25)<br>P=403	51 (8.36)<br>P=0.872
Number of leads in the system before TLE [n]	1.871±0.592<br>P=0.384	1.929±0.599<br>P<0.001	1.777±0.630<br>P=0.099
Abandoned lead before TLE	14 (8.19)<br>P=0.142	42 (13.04)<br>P=0.012	47 (7.71)<br>P=0.963
Number of leads in the heart before TLE [n]	1.965±0.719<br>P=0.104	2.084±0.699<br>P<0.001	1.861±0.703<br>P=0.129
HV lead before TLE	45 (26.13)<br>P=0.433	97 (30.12)<br>P=0.546	197 (32.30)<br>P=0.161
CS lead before TLE	30 (17.54)<br>P=0.266	43 (13.35)<br>P=0.539	92 (10.08)<br>P=0.506
Excessive lead looping due to lead fracture or ligature failure	5 (2.92)<br>P=0.403	16 (5.00)<br>P=0.127	17 (2.80)<br>P=0.868
Number of procedures before lead extraction [n]	2.229±1.281<br>P=0.418	1.906±0.938<br>P=0.050	1.849±0.949<br>P=0.015
Dwell time of oldest lead in the patient before TLE [months]	150.7±89.53<br>P<0.001	115.1±81.93<br>P=0.788	114.8±78.85<br>P<0.001
Mean implant duration before TLE [months]	142.0±81.74<br>P<0.001	104.1±69.99<br>P=0.582	108.2±71.36<br>P<0.001
Cumulative lead dwell time before TLE [years]	23.17±16.60<br>P<0.001	18.37±14.42<br>P=0.136	17.01±14.06<br>P<0.001

TLE – transvenous lead extraction, CIED – cardiac implantable electric device, SD – standard deviation, [n] – number, PM – pacemaker, AAI – single chamber pacemaker with atrial lead, VVI, VDD – single chamber pacemakers with ventricular lead, PM DDD – dual chamber pacemaker, CRT-P – cardiac resynchronization pacemaker, ICD – implantable cardioverter defibrillator, CRTD – cardiac resynchronization cardioverter defibrillator, HV lead – defibrillation leads, CS lead – coronary sinus lead

Table S2: TLE-related potential risk factors for major complications, procedure complexity and TLE efficacy and complications.

TLE-related potential risk factors for major complications, procedure complexity and TLE efficacy and complications	Flying ghosts (Ghosts shifting spontaneously to pulmonary vascular bed)	Stable ghosts (Ghosts remaining attached to cardiovascular wall)	Ghosts absent during and after TLE
Group / number of patients	1: N=171	2: N=322	3: N=610
Forms of results presentation	mean ± SD / n (%) P (1 vs. 2)	mean ± SD / n (%) P (2 vs. 3)	mean ± SD / n (%) P (1 vs. 3)
TLE-related potential risk factors for major complications and procedure complexity			
Number of extracted leads in one patient [n]	1.784 ± 0.740 P=0.778	1.795 ± 0.729 P<0.001	1.607 ± 0.680 P=0.013
Extraction of lead with excessive loop	3 (1.75) P=0.348	12 (3.73) P=0.461	16 (2.62) P=0.711
Extraction of abandoned lead(s) (any)	14 (8.19) P=0.614	41 (12.73) P=0.004	42 (6.89) P=0.678
Extraction of abandoned lead(s) [n]	0.111 ± 0.398 P<0.001	0.152 ± 0.423 P=0.256	0.084 ± 0.336 P=0.657
Longest target lead dwell time [months]	147.1 ± 88.54 P<0.001	114.5 ± 82.10 P=0.866	113.9 ± 78.63 P<0.001
Average (in the patient) target lead dwell time [months]	140.3 ± 81.83 P<0.001	106.1 ± 70.77 P=0.606	108.9 ± 71.97 P<0.001
Cumulative target lead dwell time [years]	21.79 ± 17.15 P<0.001	17.05 ± 14.68 P=0.128	15.39 ± 13.84 P<0.001
SAFeTY calculator of risk of TLE major complications [%]	3.114 ± 5.513 P=0.709	2.189 ± 2.893 P=0.016	1.960 ± 2.898 P=0.299
TLE procedure complexity			
Procedure duration (sheath-to-sheath) [minutes]	19.24 ± 31.66 P=0.126	18.85 ± 30.85 P=0.017	13.36 ± 18.07 P<0.001
Average time of single lead extraction (sheath-to-sheath) / number of extracted leads) [minutes]	9.755 ± 10.36 P=0.060	10.85 ± 21.20 P=0.735	8.205 ± 11.51 P=0.006
Technical problem during TLE (any)	53 (30.99) P=0.293	84 (26.09) P=0.399	136 (22.30) P=0.025
Number of big technical problems [n]	1.510 ± 0.982 P=0.266	1.577 ± 0.889 P=0.258	1.373 ± 0.884 P=0.060
TLE efficacy and complications			
Major complications (any)	7 (4.09) P=0.086	4 (1.24) P=0.252	16 (2.62) P=0.562
Hemopericardium	12 (7.01) P<0.001	1 (0.311) P=0.079	12 (1.97) P=0.002
Tricuspid valve damage during TLE	4 (2.34) P=0.391	3 (0.932) P=0.466	2 (0.33) P=0.033
Rescue cardiac surgery	3 (1.75) P=0.241	1 (0.311) P=0.044	14 (2.30) P=0.895

Minor complications (any)	9 (5.26)<br>P=0.277	27 (8.70)<br>P=0.371	40 (6.56)<br>P=0.661
Procedure-related death (intra-, post-procedural)	0 (0.00)<br>MN	0 (0.00)<br>MN	0 (0.00)<br>MN
Indication-related death (intra-, post-procedural	0 (0.00)<br>MN	0 (0.00)<br>MN	0 (0.00)<br>MN
Partial radiological success (remained tip or < 4 cm lead fragment)	9 (5.26)<br>P=0.270	14 (4.38)<br>P=221	16 (2.63)<br>P=0.137
Complete clinical success	166 (97.08)<br>P=0.196	319 (99.07)<br>P=0.948	606 (99.34)<br>P=0.040
Complete procedural success	159 (92.98)<br>P=0.562	305 (94.72)<br>P=0.681	592 (97.05)<br>P=0.019

TLE – transvenous lead extraction, SD – standard deviation, N – number, CVS – cardiovascular system, MN – methodologically noncomparable

Table S3: TEE findings before, during and after TLE with special focus on the presence of all forms of scar tissue and mortality after TLE

TEE findings before, during and after TLE with special focus on the presence of all forms of scar tissue and mortality after TLE	Flying ghosts (Ghosts shifting spontaneously to pulmonary vascular bed)	Stable ghosts (Ghosts remaining attached to cardiovascular wall)	Ghosts absent during and after TLE
Group / number of patients	1: N=171	2: N=322	3: N=610
Forms of results presentation	mean ± SD / n (%)<br>P (1 vs. 2)	mean ± SD / n (%)<br>P (2 vs. 3)	mean ± SD / n (%)<br>P (1 vs. 3)
Masses on lead (any)			
Scar tissue surrounding the lead	62 (36.257)<br>P=0.008	63 (19.57)<br>P<0.001	65 (10.66)<br>P<0.001
Blood clot on the lead (mobile thrombi)	31 (18.13)<br>P=0.014	32 (9.94)<br>P<0.001	23 (3.77)<br>P<0.001
Vegetation-like masses	15 (8.77)<br>P=0.050	13 (4.04)<br>P=0.026	9 (1.48)<br>P<0.001
Lead thickening	85 (49.71)<br>P=0.003	114 (35.40)<br>P=0.054	177 (29.00)<br>P<0.001
True vegetation	12 (7.02)<br>P=0.016	48 (14.91)<br>P=0.559	81 (13.28)<br>P=0.036
Lead adhesion to heart structures (any)	75 (43.86)<br>P=0.012	103 (31.99)<br>P<0.001	131 (21.48)<br>P<0.001
Lead adhesion to tricuspid apparatus	27 (15.79)<br>P=0.887	41 (12.73)<br>P=0.036	50 (8.20)<br>P<0.001
Lead adhesion to SVC	37 (21.64)<br>P<0.001	22 (6.83)<br>P=0.402	32 (5.25)<br>P<0.001
Lead adhesion to RA wall	32 (18.71)<br>P<0.004	30 (9.14)<br>P=0.112	38 (6.23)<br>P<0.001
Lead adhesion to RV wall	35 (20.470)<br>P=0.177	49 (15.22)<br>P=0.031	62 (10.16)<br>P<0.001
Lead-to-lead binding	52 (30.41)<br>P=0.146	77 (23.91)<br>P<0.001	89 (14.59)<br>P<0.001
Spread of scar tissue in CVS			
Presence of scars (thickening, floating scar, adhesion to SVC, RA, TV, RV, another lead) (all)	131 (76.61)<br>P=0.066	206 (63.98)<br>P<0.001	289 (47.46)<br>P<0.001
Number of separate scars (thickening, floating scar, adhesion to SVC, RA, TV, RV, another lead)			
Lack of any form of scar tissue	40 (23.39)<br>P=0.008	116 (36.03)<br>P<0.001	321 (52.62)<br>P<0.001
1 scar tissue manifestation	43 (25.15)<br>P=0.396	94 (29.19)<br>P=0.318	158 (25.94)<br>P=0.920
2 scar tissue manifestations	30 (17.54)<br>P=0.795	61 (18.94)<br>P=0.016	78 (12.81)<br>P=0.142
3 scar tissue manifestations	35 (20.47)	31 (9.63)	31 (5.09)

	P<0.001	P=0.012	P<0.001
4 scar tissue manifestations	13 (7.60)<br>P=0.192	14 (4.34)<br>P=0.419	17 (2.79)<br>P=0.008
5 scar tissue manifestations	6 (3.51)<br>P=0.281	5 (1.55)<br>P=0.485	5 (0.821)<br>P=0.023
6 scar tissue manifestations	3 (1.75)<br>P=0.261	1 (0.311)<br>P=0.745	0 (0.00)<br>P=0.002
TLE-related tricuspid valve dysfunction			
Increased TR by 2 degrees	3 (1.75)<br>P=0.034	21 (6.52)<br>P<0.001	3 (0.492)<br>P=0.240
Increased TR by 3 degrees	4 (2.34)<br>P=0.026	0 (0.00)<br>P=0.776	2 (0.328)<br>P=0.030
Increased TR by 2 degrees & up to grade IV	4 (2.34)<br>P=0.391	3 (0.932)<br>P=0.466	2 (0.328)<br>P=0.030
Damage to tendinous cords during TLE	7 (4.09)<br>P=0.613	18 (5.59)<br>P=0.005	12 (1.97)<br>P=0.188

TEE – transoesophageal echocardiography, TLE – transvenous lead extraction, SD – standard deviation, N / [n] – number, CVS – cardiovascular system, SVC –superior vena cava, RA – right atrium, TV – tricuspid valve, RV – right ventricle, MN – methodologically noncomparable, TR – tricuspid regurgitation
